# Deubiquitination of SARM1 by USP13 regulates SARM1 activation and axon degeneration

**DOI:** 10.1093/lifemedi/lnad040

**Published:** 2023-11-04

**Authors:** Wenkai Yue, Kai Zhang, Mingsheng Jiang, Wenjing Long, Jihong Cui, Yunxia Li, Yaoyang Zhang, Ang Li, Yanshan Fang

**Affiliations:** Interdisciplinary Research Center on Biology and Chemistry, Shanghai Institute of Organic Chemistry, Chinese Academy of Sciences, Shanghai 201210, China; University of Chinese Academy of Sciences, Beijing 100049, China; Interdisciplinary Research Center on Biology and Chemistry, Shanghai Institute of Organic Chemistry, Chinese Academy of Sciences, Shanghai 201210, China; University of Chinese Academy of Sciences, Beijing 100049, China; Interdisciplinary Research Center on Biology and Chemistry, Shanghai Institute of Organic Chemistry, Chinese Academy of Sciences, Shanghai 201210, China; University of Chinese Academy of Sciences, Beijing 100049, China; Interdisciplinary Research Center on Biology and Chemistry, Shanghai Institute of Organic Chemistry, Chinese Academy of Sciences, Shanghai 201210, China; University of Chinese Academy of Sciences, Beijing 100049, China; Interdisciplinary Research Center on Biology and Chemistry, Shanghai Institute of Organic Chemistry, Chinese Academy of Sciences, Shanghai 201210, China; Interdisciplinary Research Center on Biology and Chemistry, Shanghai Institute of Organic Chemistry, Chinese Academy of Sciences, Shanghai 201210, China; Interdisciplinary Research Center on Biology and Chemistry, Shanghai Institute of Organic Chemistry, Chinese Academy of Sciences, Shanghai 201210, China; University of Chinese Academy of Sciences, Beijing 100049, China; Key Laboratory of CNS Regeneration (Ministry of Education), Guangdong Key Laboratory of Non-human Primate Research, GHM Institute of CNS Regeneration, Jinan University, Guangzhou 510632, China; Interdisciplinary Research Center on Biology and Chemistry, Shanghai Institute of Organic Chemistry, Chinese Academy of Sciences, Shanghai 201210, China; University of Chinese Academy of Sciences, Beijing 100049, China

**Keywords:** axon injury, Wallerian degeneration, SARM1, USP13, deubiquitination

## Abstract

Sterile alpha and Toll/interleukin 1 receptor motif-containing protein 1 (SARM1) is regarded as a key protein and a central executor of the self-destruction of injured axons. To identify novel molecular players and understand the mechanisms regulating SARM1 function, we investigated the interactome of SARM1 by proximity labeling and proteomic profiling. Among the SARM1-associated proteins, we uncovered that overexpression (OE) of ubiquitin-specific peptidase 13 (USP13) delayed injury-induced axon degeneration. OE of an enzyme-dead USP13 failed to protect injured axons, indicating that the deubiquitinase activity of USP13 was required for its axonal protective effect. Further investigation revealed that USP13 deubiquitinated SARM1, which increased the inhibitory interaction between the N-terminal armadillo repeat motif (ARM) and C-terminal Toll/interleukin-1 receptor (TIR) domains of the SARM1 protein, thereby suppressing SARM1 activation in axon injury. Collectively, these findings suggest that increase of USP13 activity enhances the self-inhibition of SARM1, which may provide a strategy to mitigate axon degeneration in injury and disease.

## Introduction

In neural injury, the distal segment of an injured nerve undergoes the characteristic morphological changes, including axon beading, fragmentation, self-destruction, and engulfment and clearance by glial and immune cells, which is termed “Wallerian degeneration.” It is an active process that is tightly controlled at the molecular and cellular levels [1–3]. In 2013, sterile alpha and Toll/interleukin 1 receptor motif-containing protein 1 (SARM1) was identified as a key regulator and the first essential gene required for degeneration of injured axons [4, 5], and the loss of function of *SARM1* exerted potent axonal protection in various nerve injury models [6–8]. Later on, it was uncovered that SARM1 is a nicotinamide adenine dinucleotide (NAD^+^) hydrolase activated upon nerve injury, which rapidly depletes NAD^+^ in axons and triggers axon degeneration [5, 9–12].

**Figure 1.**
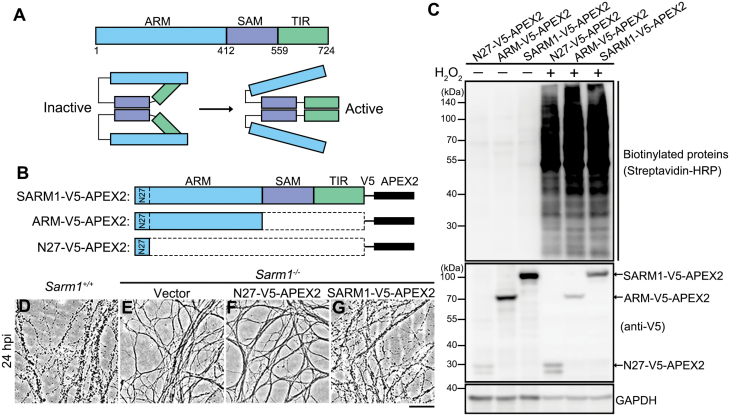
**Design and validation of proximal labeling of SARM1-interacting proteins using the APEX2 system.**(A) The schematic diagrams of the major functional domains and self-inhibition of SARM1. (B) The FL SARM1-, ARM-, and N27-V5-APEX2 constructs. (C) The expression and biotinylation efficiency in the presence of biotin-phenol and H_2_O_2_ in 293T cells were examined by Western blotting. The N27-V5-APEX2 was used as a background control for the mitochondrial localization of the transiently expressed SARM1- and ARM-V5-APEX2 (see Fig. S1). (D–G) Representative phase contrast images of axons of primary DRG neurons derived from the WT (*Sarm1*^+/+^) mice (D) or the *Sarm1* KO (*Sarm1*^−/−^) mice infected with the lentivirus to express the empty vector (E), N27-V5-APEX2 (F) or SARM1-V5-APEX2 (G) at 24 h post injury (hpi). Scale bar: 50 µm.

The major functional domains of the SARM1 protein include a C-terminal Toll/interleukin-1 receptor (TIR) domain conferring the NADase activity, two tandem sterile-alpha motif (SAM) domains that mediate the oligomerization of TIR domains, and an N-terminal armadillo repeat motif (ARM) domain that binds to the TIR domain and maintains SARM1 in the inactive state [13, 14] (Fig. 1A). Recent biochemical and structural biology studies have demonstrated that the NADase activity of SARM1 is dramatically boosted by the oligomerization of TIR domains, whereas the ARM-TIR binding prevents the TIR-TIR binding and oligomerization. Hence, the release of the ARM-TIR self-inhibition is required for SARM1 activation in Wallerian degeneration [5, 15–17]. In addition, post-translational modifications (PTMs) of the SARM1 protein such as phosphorylation by the c-jun N-terminal kinase (JNK) ( [18]) and the ratio of nicotinamide mononucleotide (NMN)/NAD^+^ [19, 20] were shown to regulate SARM1 activity. However, the exact mechanisms regulating the ARM–TIR self-inhibition in SARM1 are yet to be explored.

To identify unknown proteins participating in the regulation of SARM1, here we performed a mass spectrometry (mass spec)-based proteomic investigation of the SARM1 interactome. Among these SARM1-interacting proteins, the deubiquitinase ubiquitin-specific peptidase 13 (USP13) was found to modulate the process of SARM1-mediated axon degeneration—overexpression (OE) of the wildtype (WT) but not an enzyme-dead form of USP13 suppressed injury-induced axon degeneration, and such effect relied on the ARM-mediated inhibition of TIR dimerization. We further revealed that USP13 deubiquitinated SARM1, which increased the interaction between the ARM and TIR domains, thereby enhancing the self-inhibition of SARM1. Together, these findings suggest a previously unappreciated role and mechanism of USP13 in Wallerian degeneration, and increase of USP13 may enhance the ARM-TIR lock and attenuate SARM1 activation in nerve injury and other neurological diseases involving axon degeneration.

## Results

### Profile the SARM1 interactome by proximity labeling and mass spec analyses

To identify unknown proteins that interact with SARM1 and potentially regulate its function, we first characterized the protein–protein interaction networks of SARM1 by proximity labeling and mass spec-based proteomic analysis. The ascorbate peroxidase 2 (APEX2) [21] was fused to the C-terminus of the full-length (FL) SARM1 (SARM1-V5-APEX2) (Fig. 1B). Since SARM1 is maintained in the inactive state in intact axons and the ARM domain is required for the self-inhibition of SARM1, we hypothesized that the proteins bound specifically to the ARM domain may be of particular importance for stabilizing the ARM-TIR binding that locks SARM1 in the inactive state. To identify such proteins, we generated an ARM-V5-APEX2 construct (Fig. 1B). Additionally, the N-terminal 1−27 amino acids (N27) within the ARM domain were identified as a mitochondrial targeting sequence, which enabled a predominant mitochondrial localization of SARM1 when overexpressed in mammalian cells or axons [5, 22, 23] (Fig. S1). To exclude the non-specific labeling of mitochondrial proteins, we also generated an N27-V5-APEX2 construct as a mitochondrial localization control in the subsequent mass spec analyses (Fig. 1B).

First, we tested and verified that transient expression of the SARM1-V5-APEX2, ARM-V5-APEX2, and N27-V5-APEX2 constructs in HEK293T cells robustly and rapidly labeled cellular proteins with biotin in the presence of biotin-phenol and hydrogen peroxide (H_2_O_2_) (Fig. 1C; also see Methods). Notably, injury-induced axon degeneration was slowed down in the primary cultures of mouse dorsal root ganglia (DRG) neurons derived from the SARM1 knockout (KO) (*Sarm1*^−/−^) mice (Fig. S2A), which was reversed by lentiviral expression of the SARM1-V5-APEX2 but not the empty vector or the control construct N27-V5-APEX (Figs. 1D–G, S2B), confirming that the recombinant SARM1-V5-APEX2 protein retained the comparable axonal function of the native SARM1 and could execute axon self-destruction upon injury. Next, to demonstrate the effectiveness of the proximity labeling system, we examined a previously known SARM1-interacting protein PINK1 [23] and confirmed that it was substantially biotinylated by SARM1-V5-APEX2 compared with the vector or N27-V5-APEX2 control (Fig. S3).

**Figure 2.**
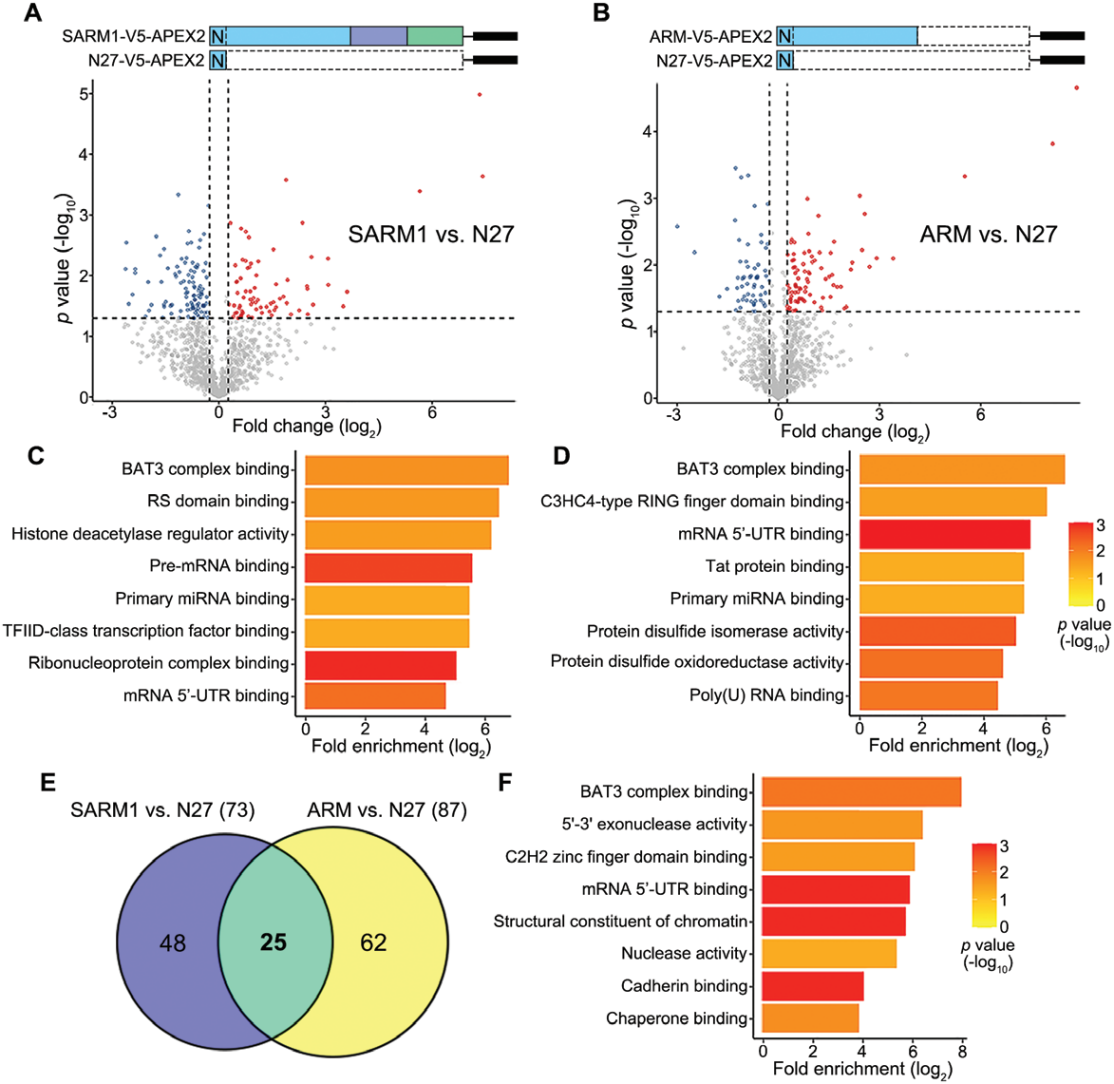
**Identification of the SARM1–ARM interacting proteins by mass spec analysis.**(A, B) Volcano plots showing the fold change (log_2_) and *p* value (−log_10_) of the SARM1- (A) or ARM- (B) interacting proteins identified in the mass spec analysis (normalized to N27-V5-APEX2). (C, D) The SARM1- (C) or ARM- (D) interacting proteins with fold change > 1.2 and *p* value < 0.05 in (A, B) are subjected to GO term analysis. The top eight terms of the molecular functions of these proteins are shown (fold enrichment, *P* value < 0.05). (E) The Venn diagram showing the proteins interacting with the FL SARM1 (73), ARM domain (87), or both (25). (F) The GO term analysis of the 25 SARM1-ARM interacting proteins (fold enrichment, *p* value < 0.05).

To characterize the SARM1 interactome, biotinylated protein lysates from the cells expressing one of the above three constructs were immunoprecipitated using the streptavidin magnetic beads. A total of 2107 proteins were recovered in the mass spec assay. Among them, 1191 proteins were detected at least twice out of 2–3 repeats in any group, which were included in the subsequent analyses (see Methods; Fig. 2A and 2B). Compared to the control (N27-V5-APEX2), 73 SARM1-interacting proteins (Table S1) and 87 ARM-interacting proteins (Table S2) were identified (fold change > 1.2 and *P* < 0.05). The Gene Ontology (GO) analysis of the SARM1- and ARM-interacting proteins showed both shared and distinct molecular functions (Fig. 2C and 2D; fold enrichment, *P* < 0.05). Of note, the SARM1-interacting proteins include not only those bound to the ARM domain but also the ones associated with the SAM or TIR domain. On the other hand, the ARM-only construct might get exposed and bind to additional proteins that do not normally interact with the ARM domain within the native SARM1. Thus, we focused on the 25 proteins that were biotin-labeled by both the FL SARM1-V5-APEX2 and ARM-V5-APEX2 constructs (Fig. 2E and 2F), as this subset of proteins were most likely to be physiologically associated with the ARM domain. Interestingly, we noticed that “BAT3 complex binding” was the most enriched term in the GO analysis of the 25 SARM1-ARM interacting proteins (Fig. 2F) as well as the SARM1- or the ARM-interacting proteins (Fig. 2C and 2D).

### USP13 interacts with SARM1 and regulates axonal integrity via SARM1

**Figure 3.**
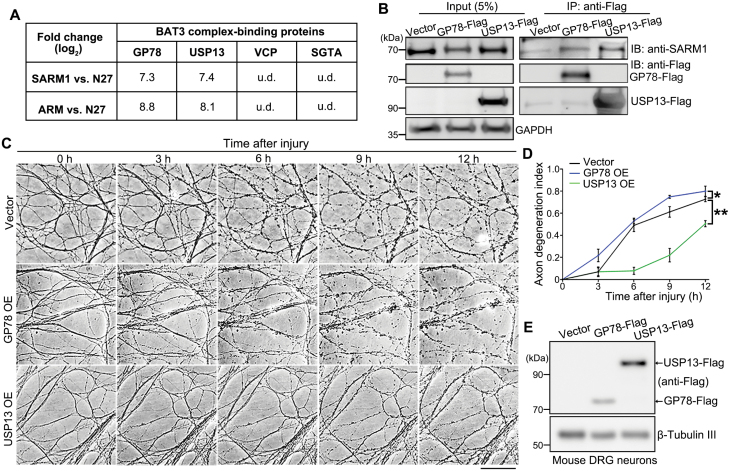
**USP13 interacts with SARM1 and regulates axonal integrity.**(A) A summary of the four proteins of the GO term “BAT3 complex binding” and the interacting intensity (fold changes in log_2_) of them in the mass spec analysis. (B) Representative images of the co-IP and Western blot assay evaluating the interaction between the endogenous SARM1 and transiently expressed GP78-Flag or USP13-Flag. (C, D) Representative images (C) and quantification (D) of injury-induced axon degeneration in the *in vitro* cultured mouse DRG neurons infected with the lentivirus expressing GP78 or USP13. Images were captured *live* at the indicated time points. (E) Western blot images confirming the heterologous expression of USP13 and GP78 in mouse DRG neurons. u.d., undetected. IB, immunoblotting. Mean ± standard error of the mean (SEM); *n* = 3. *p < 0.05, **P < 0.01; two-way ANOVA. Scale bar: 50 µm.

The proteins in the GO term of “BAT3 complex binding” include glycoprotein 78 (GP78), USP13, valosin-containing protein (VCP), and small glutamine-rich tetratricopeptide repeat-containing protein alpha (SGTA). Among them, GP78 and USP13 strongly interacted with the SARM1-ARM, whereas VCP and SGTA were undetected in the mass spec assay (Fig. 3A). GP78, an E3 ubiquitin ligase, and USP13, a deubiquitinase, reportedly both bind to and regulate the ubiquitination level of the BAT3 complex [24]. The interaction of these two proteins with the endogenous SARM1 was validated in HEK293T cells by co-immunoprecipitation (co-IP) (Fig. 3B), echoing the interactome data.

As in the experimental design of our mass spec assay, we hypothesized that the protein(s) constantly binding to the ARM domain might facilitate the ARM–TIR inhibition of SARM1, thereby preventing axon degeneration in injury. To test this hypothesis, we examined whether OE of GP78 or USP13 could exert axonal protection in the DRG axotomy model (Fig. 3C–E). In the control group, injured axons displayed remarkable degenerative features (e.g. axon beading and fragmentation) at 6 hpi, which became even more obvious at 12 hpi. GP78 OE did not inhibit injury-induced axon degeneration; instead, it slightly accelerated this process. In contrast, USP13 OE substantially delayed Wallerian degeneration, as marked axonal fragmentation was not evident until at 9 hpi and the USP13 OE group showed much better axon morphology and integrity than the vector control or GP78 OE group at 12 hpi. These data indicate that increase of USP13, but not GP78, levels is capable of suppressing injury-induced axon degeneration.

Next, we sought to determine whether the axonal function of USP13 depended on the ARM–TIR self-inhibition mechanism. To test this idea, we generated an inducible system of TIR dimerization (Fig. S4A), which used the chemically engineered ligand AP20187 to mediate the homodimeric interaction of FKBP^F36V^-TIR and subsequently elicited the NADase activity [9]. In this way, we bypassed the endogenous molecular mechanisms regulating the ARM–TIR inhibitory lock, thereby mimicking SARM1 activation without the actual injury or injury-induced signaling. If USP13 functioned by regulating the ARM-mediated inhibition of TIR dimerization, USP13 OE would not be able to suppress this FKBP^F36V^-TIR dimerization-induced axon degeneration; if USP13 inhibited axon degeneration downstream of TIR activation, USP13 OE would still be axonal protective in this setting. The results showed that, unlike injury-induced axon degeneration (Fig. S4B), USP13 OE failed to suppress FKBP^F36V^-TIR dimerization-triggered axon degeneration (Fig. S4C). These results demonstrate that the axonal protective effect of USP13 requires and acts upstream of the ARM-mediated inhibition of TIR dimerization and/or activation.

### USP13 deubiquitinates SARM1 and the deubiquitinase activity is required for axonal protection

**Figure 4.**
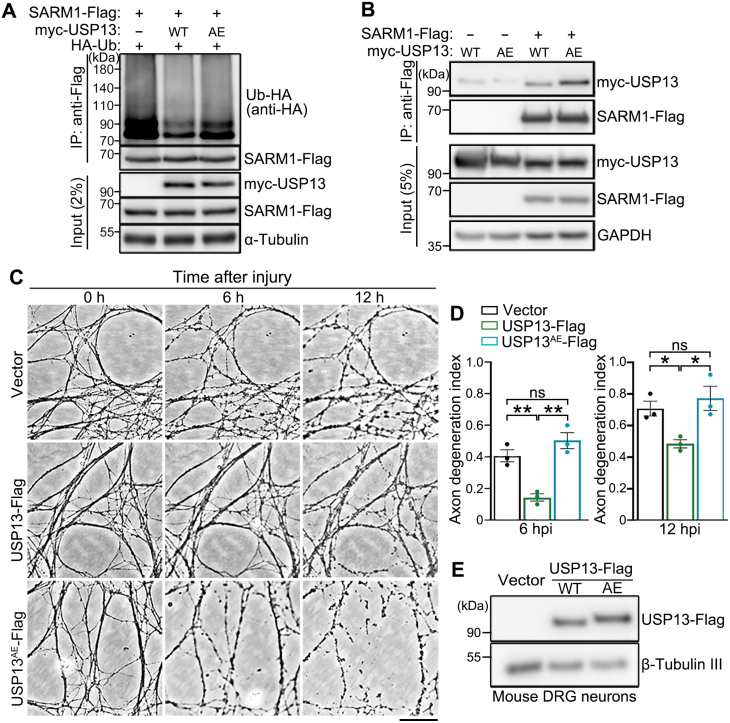
**USP13 deubiquitinates SARM1 and its axonal protective effect requires the deubiquitinase activity.**(A, B) Representative Western blot images showing the ubiquitination levels of SARM1 in HEK293T cells co-expressed with WT USP13 or USP13^AE^ (A) and the interaction between them and SARM1 (B). (C, D) Representative phase contrast images (C) and quantification (D) of injury-induced axon degeneration in primary mouse DRG neurons infected with the lentivirus expressing WT USP13 or USP13^AE^. Images were captured live at the indicated time points after axotomy. (E) Western blot assay confirming the lentiviral expression of WT USP13 or USP13^AE^ in the *in vitro* cultured mouse DRG neurons. hpi, hours post injury. Mean ± SEM; *n* = 3. *p < 0.05, **p < 0.01; ns, no significance; one-way ANOVA. Scale bar: 50 µm.

USP13 is a deubiquitinating enzyme involved in a myriad of cellular processes, such as energy metabolism, DNA damage response, endoplasmic reticulum-associated degradation, and autophagy by regulating the ubiquitination levels of different protein substrates [25–27]. To test whether USP13 regulated SARM1 ubiquitination, we overexpressed USP13 in HEK293T cells, which dramatically decreased SARM1 ubiquitination levels (Fig. 4A). In contrast, OE of GP78 did not significantly affect SARM1 ubiquitination levels (Fig. S5), which was consistent with the observation that OE of GP78 did not manifest axonal protection in nerve injury (Fig. 3D).

The USP13^AE^ mutation (C345A/M644/739E) was previously shown to substantially attenuate the deubiquitinase activity of USP13 [28]. Indeed, OE of USP13^AE^ mutation compromised the deubiquitinating function of USP13 on SARM1 (Fig. 4A), despite that the USP13^AE^ mutation showed an enhanced binding to SARM1 compared to the WT USP13 protein (Fig. 4B). In mouse DRG neurons, we showed that OE of WT USP13 but not the enzymatically inactive USP13^AE^ mutation delayed injury-induced axon degeneration (Fig. 4C–E). Together, the results indicate that the deubiquitinase activity of USP13 is required for its axonal protective function. Of note, although ubiquitination is commonly associated with the regulation of protein turnover, deubiquitination of SARM1 by USP13 did not show any noticeable effect on the protein levels of SARM1 (Fig. 4A and 4B). Thus, the axonal protective effect of USP13 OE was unlikely due to a change of SARM1 abundance but rather by regulating its activation.

We noticed that USP13 OE not only reduced SARM1 ubiquitination levels but also decreased the overall protein ubiquitination levels (Fig. S6A). It raised the possibility that reduction of overall protein ubiquitination levels might manifest axonal protection. To test this idea, we introduced another deubiquitination enzyme of the ubiquitin-specific peptidase (USP) family [29], USP10, which shares some substrates with USP13 and participates in regulating the same cellular functions such as the initiation of autophagy [30]. We found that USP10 OE markedly reduced the overall protein ubiquitination levels, but it did not affect SARM1 ubiquitination levels (Fig. S6B). Nor did USP10 interact with SARM1 examined by the co-IP experiment (Fig. S6C). More important, unlike USP13, OE of USP10 was unable to protect injured axons (Fig. S6D–F), indicating that it is the specific deubiquitination of SARM1 by USP13 but not merely a reduction of the overall protein ubiquitination levels that yields the axonal protection.

### Deubiquitination by USP13 enhances ARM–TIR interaction

As mentioned above, USP13 regulated SARM1 activation and ubiquitination without affecting its protein abundance (Fig. 4). We were keen to understand how USP13 regulates SARM1 activation. The ARM–TIR interaction is key to the self-inhibition of SARM1, as deletion of the ARM domain or disrupting the ARM–TIR interfaces activates SARM1 and causes axon degeneration [5], whereas increasing the ARM–TIR interaction exerted axonal protection following injury [31]. Hence, we examined how USP13 levels affected the ARM–TIR interaction with the co-IP assay.

**Figure 5.**
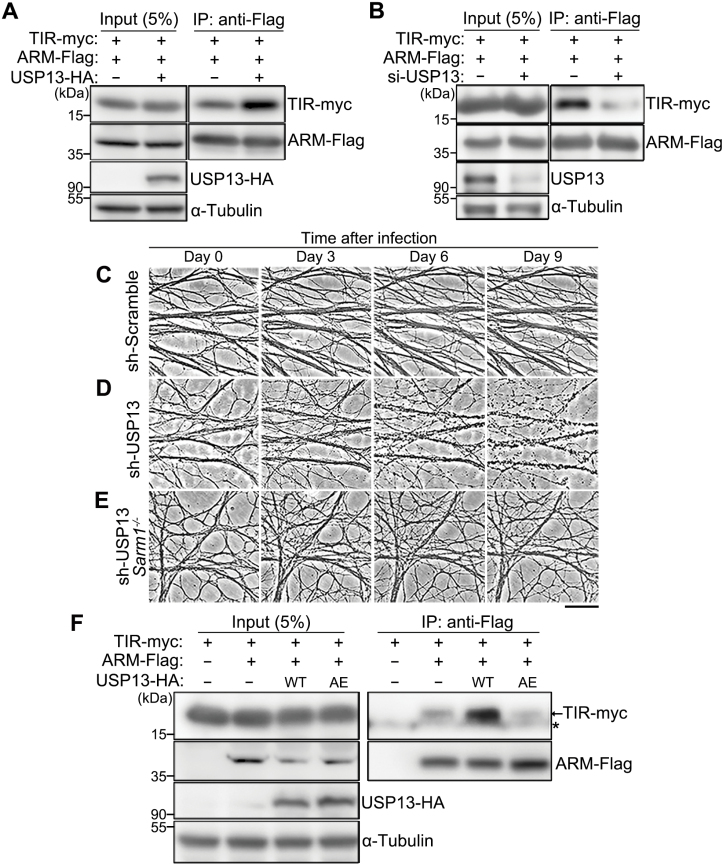
**USP13 regulates the ARM–TIR interaction by deubiquitination.**(A, B) Representative images of the co-IP assay showing the interaction between the ARM and TIR domains in HEK293T cells, with USP13 OE (A) or USP13 KD (B). The siRNA cocktail containing three independent oligonucleotides (1:1:1) targeting human USP13 is used for USP13 KD (also see Methods and Table S3). (C–E) Representative phase contrast images of axons of *in vitro* cultured mouse DRG neurons infected with the lentivirus expressing the scramble shRNA (sh-Scramble) (C) or the shRNA against USP13 (sh-USP13) in the WT (D) or SARM1 KO (*Sarm1*^*−*/*−*^) (E) background. Images are captured *live* at the indicated time points after lentiviral infection. Scale bar: 50 µm. (F) The co-IP assay indicating the interaction between the ARM and TIR domains is increased by OE of WT but not the inactive mutant (AE) of USP13. The asterisk (*) represents a non-specific band.

We found that USP13 OE remarkably enhanced the ARM–TIR interaction (Fig. 5A), whereas USP13 knockdown (KD) had the opposite effect (Fig. 5B). Concurrent with the reduced ARM–TIR interaction, KD of USP13 in mouse DRG neurons led to spontaneous axon degeneration without injury (Fig. 5C and 5D). And, the USP13 KD-induced axon degeneration depended on the function of SARM1 as the SARM1 KO (*Sarm1*^−/−^) completely rescued the spontaneous axon degeneration of USP13 KD (Fig. 5D and 5E). Furthermore, we showed that the enzymatically inactive UAP13^AE^ mutation could not promote the ARM–TIR interaction as the WT USP13 (Fig. 5F). Collectively, our findings indicate that the deubiquitinase USP13 reduces SARM1 ubiquitination levels and promotes the ARM–TIR interaction, thereby enhancing the self-inhibition of SARM1 and suppressing axon degeneration in nerve injury.

## Discussion

**Figure 6.**
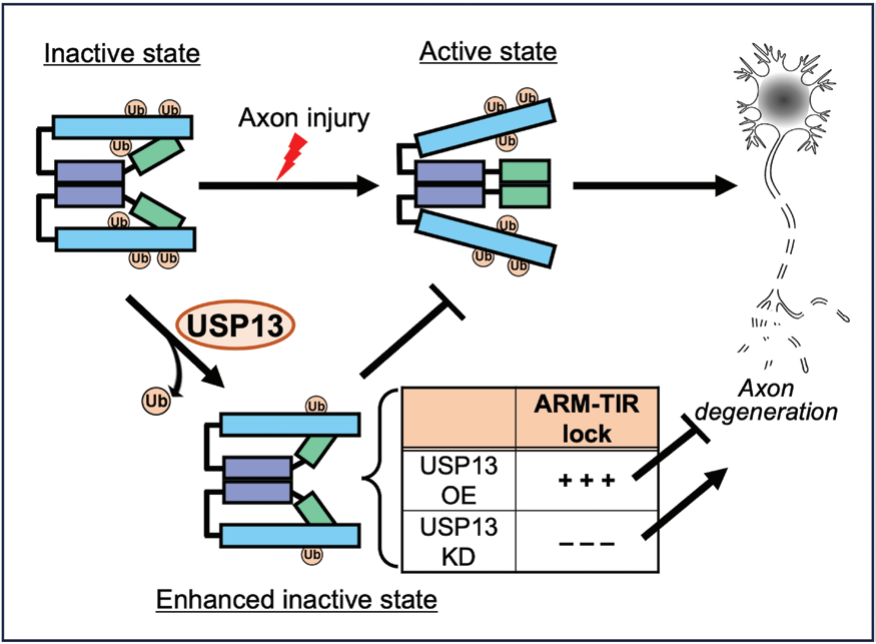
**A schematic model of the role and mechanism of USP13 in regulating SARM1 self-inhibition in Wallerian degeneration.**In intact axons, SARM1 is maintained in the inactive state by the self-inhibition mediated by the ARM–TIR lock. After axon injury, the ARM–TIR interaction is unlocked, triggering SARM1 activation and the subsequent axon degeneration. The deubiquitinase USP13 reduces SARM1 ubiquitination levels, which increases ARM–TIR interaction and intensifies the SARM1 self-inhibition. As such, USP13 OE enhances the ARM–TIR lock and suppresses injury-induced axon degeneration; whereas USP13 KD diminishes the ARM–TIR inhibition, leading to spontaneous axon degeneration without injury. Ub, ubiquitin; OE, overexpression; KD, knockdown.

In this study, we identified USP13 as a novel regulator of SARM1-mediated axon degeneration by means of a mass spec-based analysis of the SARM1 interactome. USP13 OE delays injury-induced axon degeneration and the deubiquitinase activity of USP13 is required for this axonal protective effect. We further reveal that USP13 reduces SARM1 ubiquitination levels and enhances the ARM–TIR interaction. Together, our findings indicate that deubiquitination of SARM1 by USP13 strengthens the ARM–TIR self-inhibition, thereby enhancing the inactive state of SARM1 and attenuating axon degeneration in injury (Fig. 6).

A variety of PTMs including phosphorylation, ubiquitination, palmitoylation and acetylation regulate axon degeneration. For instance, phosphorylation by glycogen synthase kinase 3β (GSK3β) triggers microtubule reorganization and promotes autophagy in injured axons [32, 33], and a mitogen-activated protein kinase cascade transduces the injury signal to axon self-destruction via phosphorylation [34]. Meanwhile, phosphorylation of SARM1 at S548 regulates its NADase activity [18, 35]. The ubiquitin-proteasome system (UPS) is a central machinery for protein degradation, and ubiquitination-dependent mechanisms play a crucial role in maintaining axonal function and integrity [36, 37]. Particularly, several E3 ubiquitin ligases and the complex components regulating the turnover of nicotinamide mononucleotide adenylyltransferas (NMNAT), a major axonal protective protein, are identified [38–41]. In addition, palmitoylation also regulates protein degradation of NMNAT2 and other proteins in axons, thereby modulating the progression of Wallerian degeneration [42–44]. Besides, deacetylation of microtubule reduces the stability of axonal cytoskeleton and exacerbates injury-induced axon degeneration [45].

Despite the well-known function of ubiquitination in regulating protein turnover, our data clearly show that deubiquitination of SARM1 by USP13 does not significantly alter the protein abundance of SARM1. Instead, deubiquitination of SARM1 by USP13 enhances the ARM–TIR interaction and stabilizes this self-inhibition lock. Consistently, we find that KD of USP13 increases SARM1 ubiquitination levels, which reduces the ARM–TIR interaction likely due to the steric hindrance by ubiquitin, leading to SARM1 activation and spontaneous axon degeneration. Thus, our current study provides the first evidence that ubiquitination of SARM1 regulates its activation in axon degeneration.

Recent studies show that the relative NMN/NAD^+^ levels control SARM1 activation through the allosteric regulation of the ARM domain—the NMN-binding ARM domain shifts from an inhibitory state to a permissive state, releasing the TIR domains for oligomerization that substantially boosts the NADase activity [19, 20]. In the future, it will be interesting to investigate whether ubiquitination of SARM1 occurs in the ARM or TIR domain and whether deubiquitination of SARM1 by USP13 affects the binding and/or allosteric regulation of NMN/NAD^+^. Nevertheless, our findings demonstrate a role and mechanism of the deubiquitinase USP13 in regulating SARM1-mediated axon degeneration, which may shed insight on the development of novel therapeutic strategies to suppress axon degeneration in injury and diseases.

### Research limitations

While our work identifies USP13 as a deubiquitinase of SARM1 and highlights that USP13 decreases SARM1 ubiquitination levels and delays Wallerian degeneration by enhancing the inhibitory ARM–TIR interaction, the above-mentioned evidence has been derived exclusively from *in vitro* systems, such as mouse primary neuron cultures and human cell lines. Thus, validating the axonal function of USP13 in an *in vivo* model is desirable in the future. In addition, due to lack of the proper antibodies, whether the protein levels of USP13 or the ubiquitination levels of SARM1 in mouse DRG axons change upon injury is to be defined. Meanwhile, the current data do not rule out the possibility that the axonal function of USP13 involves other protein substrates or cellular functions in addition to its regulation of SARM1 activation. Altogether, further investigation is warranted to better understand the mechanism and therapeutic potential of USP13 in nerve injury and axon degeneration.

## Methods

### Plasmids and constructs

The pCMV-HA-Ub (Z. Zhang), pLenti-hSyn [46], and pCAG-hPINK1-V5 plasmids [47] were kind gifts. To generate pcDNA3.1-SARM1-Flag, pcDNA3.1-USP13-Flag, pcDNA3.1-GP78-Flag, pcDNA3.1-GP78-myc, and pcDNA3.1-myc-USP10 plasmids, the DNA fragments encoding human *SARM1*, *USP13*, *GP78*, and *USP10* were amplified from the cDNA of HEK293T cells by polymerase chain reaction (PCR) with specific primers (Table S3), and then inserted into the pcDNA3.1 expression vector (SnapGene) between the *Bam*HI and *Eco*RI sites using the ClonExpress MultiS One Step Cloning Kit (Vazyme; C113).

To generate the pcDNA3.1-N27-V5-APEX2, pcDNA3.1-SARM1-V5-APEX2, pcDNA3.1-ARM-V5-APEX2, pcDNA3.1-ARM-Flag, pcDNA3.1-TIR-myc, pcDNA3.1-myc-USP13, pcDNA3.1-USP13-HA, pcDNA3.1-myc-USP13^AE^ (C345A/M644/739E), and pcDNA3.1-USP13^AE^-HA plasmids, the truncated *SARM1* fragments were amplified from the FL *SARM1* sequence with the designated primers (Table S3), the APEX2 fragment was amplified from pCAG-APEX2-BACE1-HA plasmid (a kind gift from Y. Chen), and the USP13^AE^ mutant was generated by homologous recombination of DNA fragments containing the desired point mutations derived from WT USP13 by PCR, and inserted into the pcDNA3.1 vector using the method as described above. The empty pcDNA3.1 vector was used as a control in the transfection experiments involving pcDNA3.1-derived constructs.

To generate the lentiviral vectors including the pLenti-hSyn-N27-V5-APEX2, pLenti-hSyn-SARM1-V5-APEX2, pLenti-hSyn-USP13-Flag, pLenti-hSyn-USP13^AE^-Flag, pLenti-hSyn-GP78-Flag, and pLenti-hSyn-Flag-USP10, the DNA fragments were amplified from the corresponding pcDNA3.1 plasmids and subcloned into the pLenti-hSyn vector using the *Bam*HI/*Eco*RI sites. To generate the pLenti-hSyn-myc-FKBP^F36V^-TIR plasmid, the DNA fragment encoding human *FKBP* was amplified from the cDNA of HEK293T cells by PCR, and the F36V mutation was introduced as described above. The FKBP^F36V^ and TIR fragments were then ligated into the pLenti-hSyn vector using the *Bam*HI/*Eco*RI sites. The empty pLenti-hSyn vector was used as a control in the transfection experiments involving pLenti-hSyn-derived constructs.

For the pLKO.1-shUsp13 plasmid, the shRNA of the mouse Usp13 gene was cloned into the pLKO.1 vector using T4 DNA ligase (Thermo Fisher Scientific; EL0011). The control vector containing scrambled shRNA was obtained from Addgene (no. 1864).

The siRNA cocktail targeting human USP13 included three different oligonucleotides (1:1:1). All siRNAs including the scrambled siRNA control were purchased from GenePharma (Shanghai, China). The sequences of all the primers and siRNAs used in this study are summarized in Table S3.

### Cell cultures and transfection

HEK293T cells (American Type Culture Collection; CRL-11268) were cultured in DMEM (Sigma-Aldrich; D0819) supplemented with 10% (vol/vol) fetal bovine serum (Biowest; S1710) at 37°C in 5% CO_2_. Transient transfection of siRNA oligonucleotides or plasmids was performed using Lipofectamine RNAiMAX (Invitrogen; 13778075) in Opti-MEM (Invitrogen; 11058021) or the *in vitro* DNA Transfection Reagent PolyJet (SignaGen Laboratories; SL100688) in DMEM. Cells were harvested at 36 h and 72 h after transfection with the expression plasmids and siRNAs, respectively.

### Biotin labeling with APEX2 and sample preparation for mass spec

Cells expressing the APEX2 constructs were treated with 500 mM biotin-phenol for 30 min. Prior to harvesting, biotinylation of proteins was catalyzed by addition of 1 mM H_2_O_2_ for 1 min, and the reaction was quenched by washing cells twice with the phosphate-buffered saline (PBS) containing 10 mM sodium ascorbate and 5 mM Trolox. Each group comprised 2–3 repeats and the cell lysates were collected for the IP enrichment. Briefly, HEK293T cells were lysed in the RIPA buffer (50 mM Tris–HCl at pH 7.4, 150 mM NaCl, 0.1% SDS, 1% NP-40, 1 mM EDTA, 5% glycerol) containing the protease inhibitor cocktail (Roche; 5892791001). Following centrifugation at 15,000 × *g* for 15 min at 4°C, the supernatants were collected in new vials, and incubated with streptavidin magnetic beads (Thermo Fisher Scientific; 88816) on a rotary shaker at 4°C overnight. The beads were then collected for the subsequent mass spec sample preparation.

For each IP sample containing beads, 50 μL of 8 M urea in the Tris–HCl buffer (100 mM, pH 8.0) were added for denaturation. Thereafter, the proteins were reduced with 5 mM Tris(2-carboxyethyl)phosphine (TCEP) for 20 min at room temperature and alkylated with 10 mM iodoacetamide (IAA) for 15 min in the dark. The urea concentration was then adjusted to 2 M, and 1 mM CaCl_2_ and 1 μg trypsin were further added for protein digestion at 37°C overnight. Digested peptides were desalted with C18-tips for the subsequent mass spec analysis.

### Liquid chromatography-tandem mass spectrometry (LC–MS/MS) analysis

The LC–MS/MS analysis was performed using an on-line EASY-nLC 1000 HPLC coupled with an Orbitrap Fusion mass spectrometer (Thermo Fisher Scientific). The peptide mixture was separated by an in-house manufactured 15-cm capillary column packed with the C18 resin (1.9 μm, Dr. Maisch GmbH) at a flow rate of 300 nL/min. Mass spectra were acquired in a data-dependent mode with one full scan (m/z: 350–1500; resolution: 120,000; AGC target value: 1,000,000), followed by MS2 scan (32% normalized collision energy; AGC target value: 100,000; maximal injection time: 45 ms).

### LC–MS/MS data processing

The protein identification and quantification were performed by MaxQuant (version 2.0.1.0). The precursor mass tolerance and the fragment mass tolerance were set to 20 ppm and 0.1 Da, respectively. The cysteine carbamidomethylation (delta mass = 57.021) served as a static modification, while the methionine oxidation (delta mass = 15.995) as a variable modification. The false discovery rates at the peptide spectral match level and the protein level were both controlled below 1%. The LFQ intensities were used for protein quantification. After filtering out the contaminants, a total of 2,107 proteins were detected. These proteins were first grouped according to their treatments and further filtered, requiring at least 2 valid values out of 2–3 repeats in any group. The remaining 1191 proteins were subjected to the subsequent analyses. In brief, the bioinformatics analysis was performed with Perseus (version 2.0.7.0) [48]. Following the log_2_ transformation, missing values were replaced by random numbers that were drawn from a normal distribution with a downward shift of 1.8 *σ* and a width of 0.3 to allow statistical testing.

### Protein extraction and Western blotting

Cultured cells were lysed in the lysis buffer (100 mM Tris–HCl, pH 6.8, 2% SDS, 40% glycerol, 10% β-mercaptoethanol, and 0.04% bromophenol blue) supplemented with the protease inhibitor cocktail (Roche). All protein samples were boiled at 99°C for 10 min. Equal amounts of lysates were resolved by electrophoresis using SDS-PAGE and transferred to the 0.45-µm Immobilon-P polyvinylidene fluoride membranes (Merck Millipore; IPVH00010), followed by incubation with the primary and secondary antibodies. Detection was performed using the High-sig ECL Western Blotting Substrate (Tanon; 180-5001). Images were captured by the Amersham Imager 600, with the densitometry measured by ImageJ (NIH).

### IP and co-IP

HEK293T cells were lysed in either the RIPA buffer containing both the protease inhibitor cocktail and N-Ethylmaleimide (Sigma-Aldrich; E3876) for IP, or the co-IP buffer (50 mM Tris−HCl at pH 7.4, 150 mM NaCl, 1% NP-40, 1 mM EDTA, 5% glycerol) containing the protease inhibitor cocktail for co-IP. Following centrifugation at 15,000 × g for 15 min at 4°C, the supernatants transferred into new vials were incubated with the mouse anti-Flag beads (Sigma-Aldrich; M8823) on a rotary shaker at 4°C overnight. The beads were then collected and the attached proteins were eluted with the 2 × SDS buffer for the subsequent Western blotting assays.

### Immunocytochemistry

HEK293T cells grown on chamber slides (Lab-Tek) were transfected with the indicated plasmids for 36 h, and then sequentially incubated with fresh 4% paraformaldehyde (Sangon; A500684) in the PBS (15 min), 0.5% Triton X-100 (Sigma-Aldrich; T8787) in the PBS (PBST; 20 min), and 3% goat serum in the PBST (the blocking buffer; 60 min) at room temperature. Thereafter, cells were probed with the specified antibodies in the blocking buffer at 4°C overnight, followed by incubation of the appropriate secondary antibodies for 2 h at room temperature. The chamber slides were then disassembled according to the manufacturer’s instruction and mounted in VECTASHIELD Mounting Medium with DAPI (Vector Laboratories; H-1200).

### Antibodies

The following primary antibodies were used in this study: rabbit anti-HA (CST; 3724S), mouse anti-Flag (Sigma-Aldrich; F3165), rabbit anti-myc (Sigma-Aldrich; C3956), mouse anti-GAPDH (Proteintech; 60004-1-Ig), rabbit anti-α-Tubulin (Thermo Fisher Scientific; PA5-58711), rabbit anti-β-Tubulin III (Sigma-Aldrich; T2200), rabbit anti-SARM1 (CST; 13022), mouse anti-V5 (Proteintech; 66007-1-Ig), mouse anti-USP13 (Santa Cruz; sc-514416), and rabbit anti-PINK1 (Novus Biologicals; BC100-494). The following HRP-conjugated secondary antibodies were used: goat anti-mouse IgG (Sigma-Aldrich; A4416) and goat anti-rabbit IgG (Sigma-Aldrich; A9169). The fluorophore-conjugated secondary antibody used was goat anti-mouse Alexa Fluor 488 (Life Technologies; A11029).

### Lentiviral vectors and infection

To generate lentivirus for infecting primary neurons, HEK293T cells were co-transfected with pLenti or pLKO.1, psPAX2 and pMD2.G plasmids at a ratio of 4:2:1 in DMEM using PolyJet. The supernatant of the cell culture was collected at 48 h after transfection and passed through a 0.45-µm filter (Merck Millipore; SLHVR33RB). Fresh supernatants containing viral particles for infection were used immediately or stored at 4°C for less than a week.

### DRG neuron culture, in vitro axotomy and quantification of axon degeneration

Mouse DRG neurons were dissected at embryonic days 13.5 or 14.5 from WT or *Sarm1*^−/−^ (Jackson Laboratory, Jax no. 018069 [49] mouse embryos in a C57BL/6 background. Isolation of embryonic DRG was conducted as previously described [50]. Dissected DRG were digested with a mixture of collagenase and TrypLE Express Enzyme (Gibco; 12604013), and a drop (5 µL) of the DRG neuron suspension was placed onto the center of a 12-well plate for 15–20 min. After cells were firmly attached, 1 mL of the neurobasal medium (Gibco; 21103049) supplemented with 2% B27 (Gibco; 17504044) and 1% GlutaMAX (Invitrogen; 35050061) was gently added to the well. The DRG neurons were then infected on 7 days *in vitro* (DIV), and axotomy was performed under the microscope using a micro surgical blade (WHB; C21) on 14 DIV.

Phase contrast images of *in vitro* cultured DRG neurites were taken *live* at the indicated time points after axotomy with a Leica DMi8 inverted microscope. The severity of neurite degeneration was evaluated by the degeneration index calculated as the ratio of the pixel number of degenerated neurites to that of total neurites [32]. Numbers of random, nonoverlapping images of different DRG explants of each group from pooled results of at least three independent repeats are indicated in each figure.

### Research ethics

All surgical procedures in mice were performed in compliance with the institutional guidelines on the scientific use of living animals at Interdisciplinary Research Center on Biology and Chemistry, the Chinese Academy of Sciences. Meanwhile, “Principles of laboratory animal care” (NIH publication No. 86-23, revised 1985) was also followed in the current study. Animal distress and conditions requiring euthanasia were addressed and the number of animals used was minimized.

### Statistical analysis

Statistical significance in this study is determined by one-way analysis of variance (ANOVA) with Tukey’s honestly significant difference post hoc test, two-way ANOVA with Bonferroni’s post hoc test, or unpaired, two-tailed Student’s *t*-test with equal variance (*P < 0.05; **P < 0.01; ***P < 0.001), as indicated. Error bars represent the SEM.

## Data availability

All data that support the conclusions of this study are presented in the main manuscript and its supporting materials.

## Supplementary Material

lnad040_suppl_Supplementary_Figures_S1-S6

lnad040_suppl_Supplementary_Tables_S1

lnad040_suppl_Supplementary_Tables_S2

lnad040_suppl_Supplementary_Tables_S3
