# Supplementary material for: Deubiquitination of SARM1 by USP13 regulates SARM1 activation and axon degeneration
Source: Life Med. 2023 Nov 4;2(5):lnad040. doi: 10.1093/lifemedi/lnad040 (PMC11749472; doi:10.1093/lifemedi/lnad040)
Supplement: lnad040_suppl_Supplementary_Figures_S1-S6 [file lnad040_suppl_Supplementary_Figures_S1-S6.pdf]

SUPPLEMENTAL INFORMATION

Deubiquitination of SARM1 by USP13 regulates SARM1 activation and axon degeneration

Wenkai Yue, Kai Zhang, Mingsheng Jiang, Wenjing Long, Jihong Cui, Yunxia Li, Yaoyang Zhang, Ang Li and Yanshan Fang

Correspondence to: anglijnu@jnu.edu.cn (A.L.); fangys@sioc.ac.cn (Y.F.)

Supplemental Inventory

1. Supplemental Figures

Figure S1, related to Figure 1

Figure S2, related to Figure 1

Figure S3, related to Figures 1 and 2

Figure S4, related to Figure 3

Figure S5, related to Figure 4

Figure S6, related to Figure 4

2. Supplemental Tables (in separate spreadsheets)

Table S1, related to Figure 2

Table S2, related to Figure 2

Table S3, related to Figures 1-5

SUPPLEMENTAL FIGURES

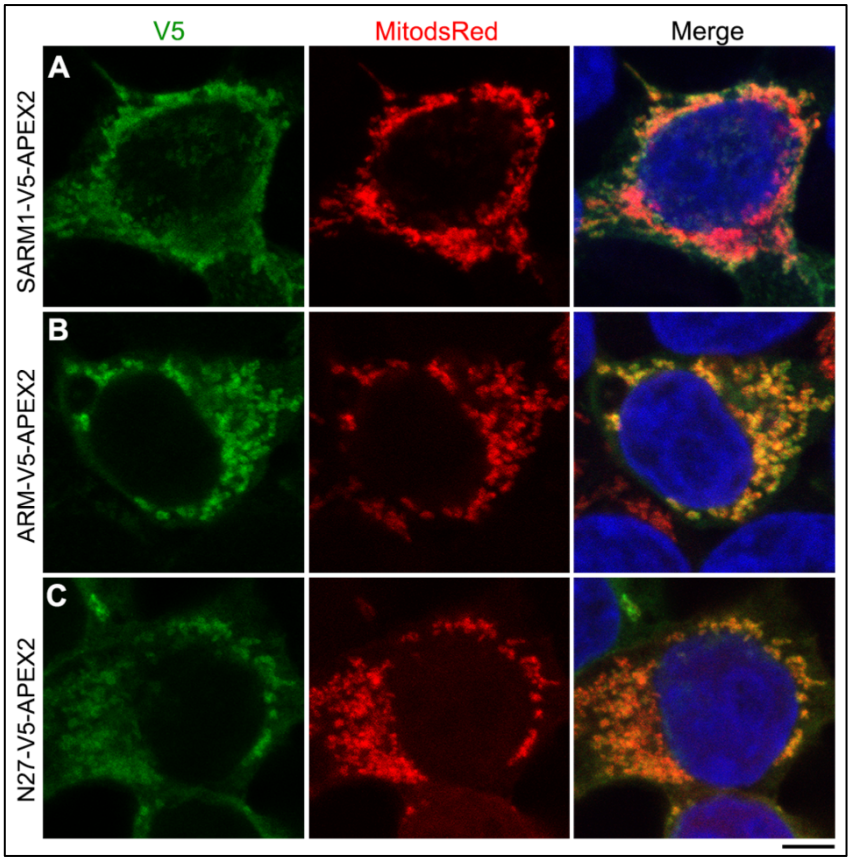

Figure S1. Expression of the three SARM1-APEX2 constructs in 293T cells

(**A-C**) Representative confocal images of 293T cells transiently expressing the full-length SARM1- (A), ARM- (B) or N27-V5-APEX2 (C) with the antibody against the V5 tag, which confirms the predominant mitochondrial localization of these recombinant proteins. The blue color shows DAPI staining (for the nucleus); MitodsRed, indicating mitochondria. Scale bar: 5 μm.

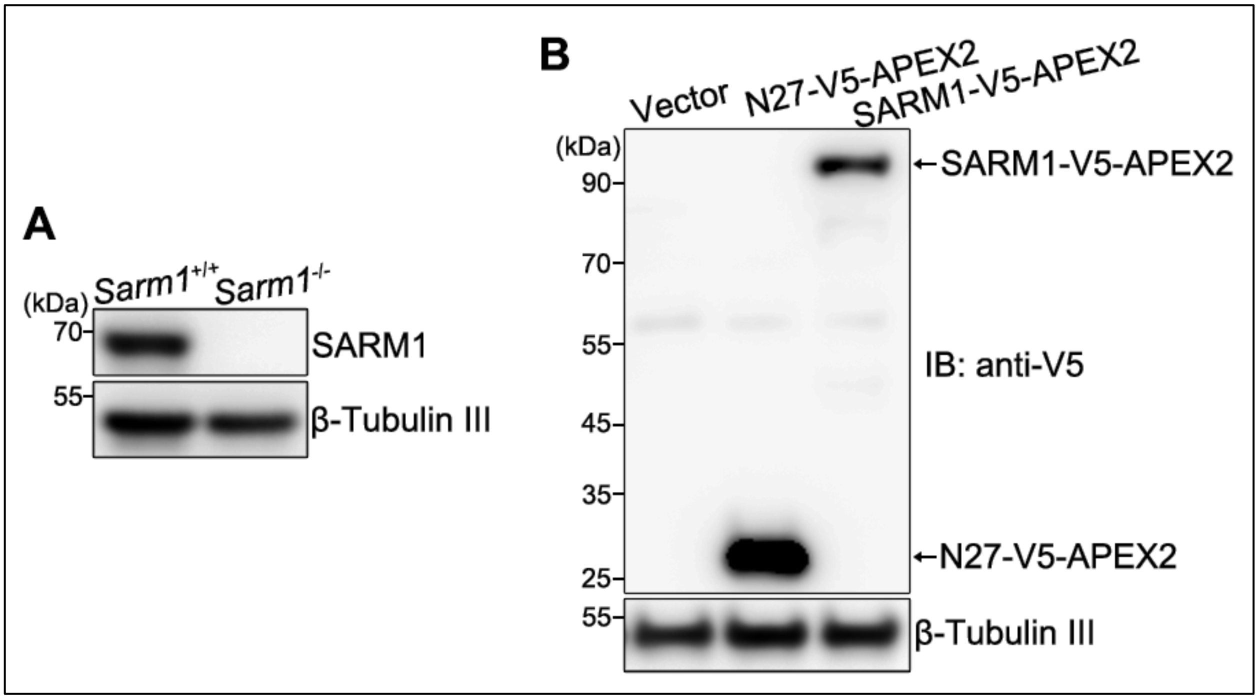

Figure S2. Validation of the SARM1 KO and expression of the APEX2 constructs

(**A**) The western blot assay showing the protein levels of SARM1 in the WT (*Sarm1*$^{+/+}$) or SARM1 KO (*Sarm1*$^{-/-}$) mouse DRG neurons. (**B**) The expression of the empty vector, N27-V5-APEX2 or SARM1-V5-APEX2 is confirmed by western blot with the anti-V5 antibody.

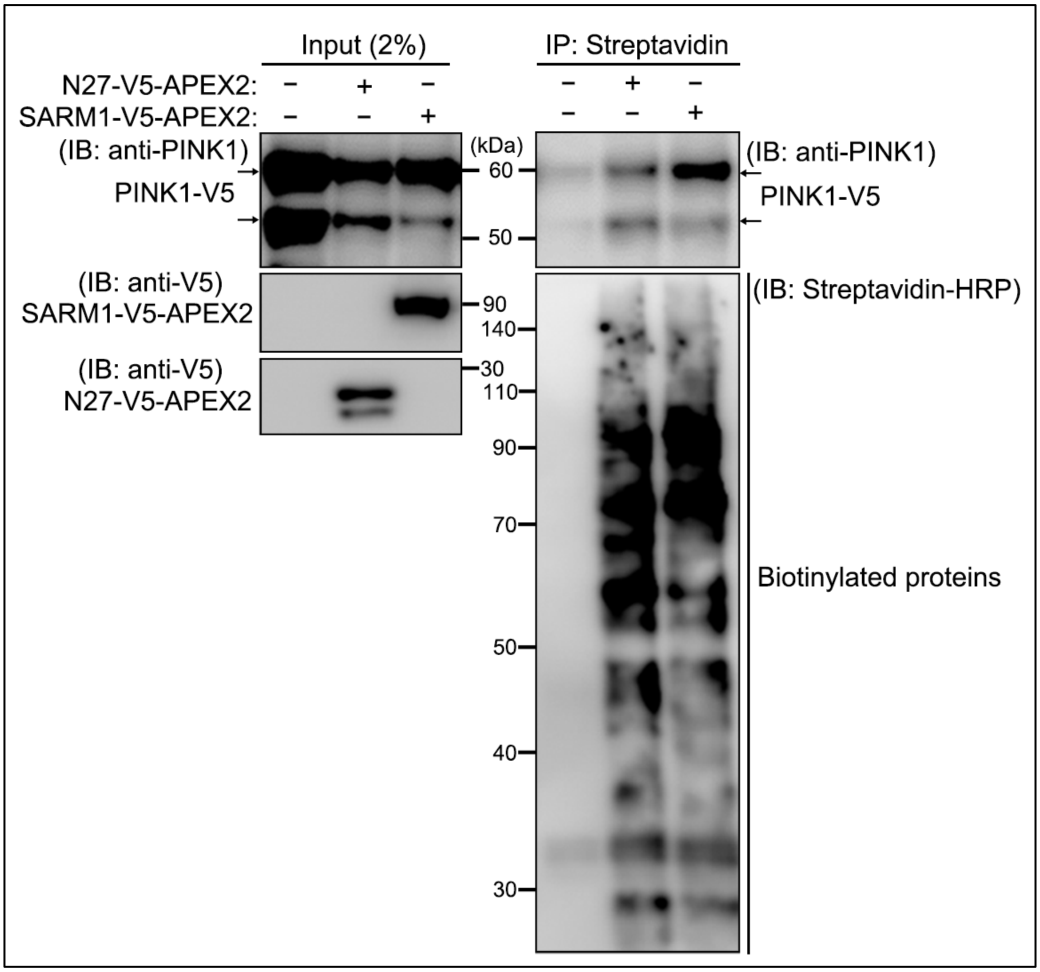

Figure S3. Confirmation of biotin labeling of the known SARM1-associated protein PINK1

Representative western blot images showing the expression of PINK1-V5 (endogenous PINK1 protein is undetectable without mitochondrial stress) in 293T cells co-transected with the empty vector, N27-V5-APEX2, or SARM1-V5-APEX2 (2% input; on the left). The cells are treated with biotin-phenol and H_2O_2 (see Materials and Methods), and then immunoprecipitated (IP) using the streptavidin-beads and immunoblotted (IB) with anti-PINK1 for biotinylated PINK1-V5 or streptavidin-HRP for all biotinylated proteins (on the right). Substantially more PINK1-V5 proteins are labeled with biotin in the SARM1-V5-APEX2 group compared to the empty vector or the N27-V5-APEX2 group.

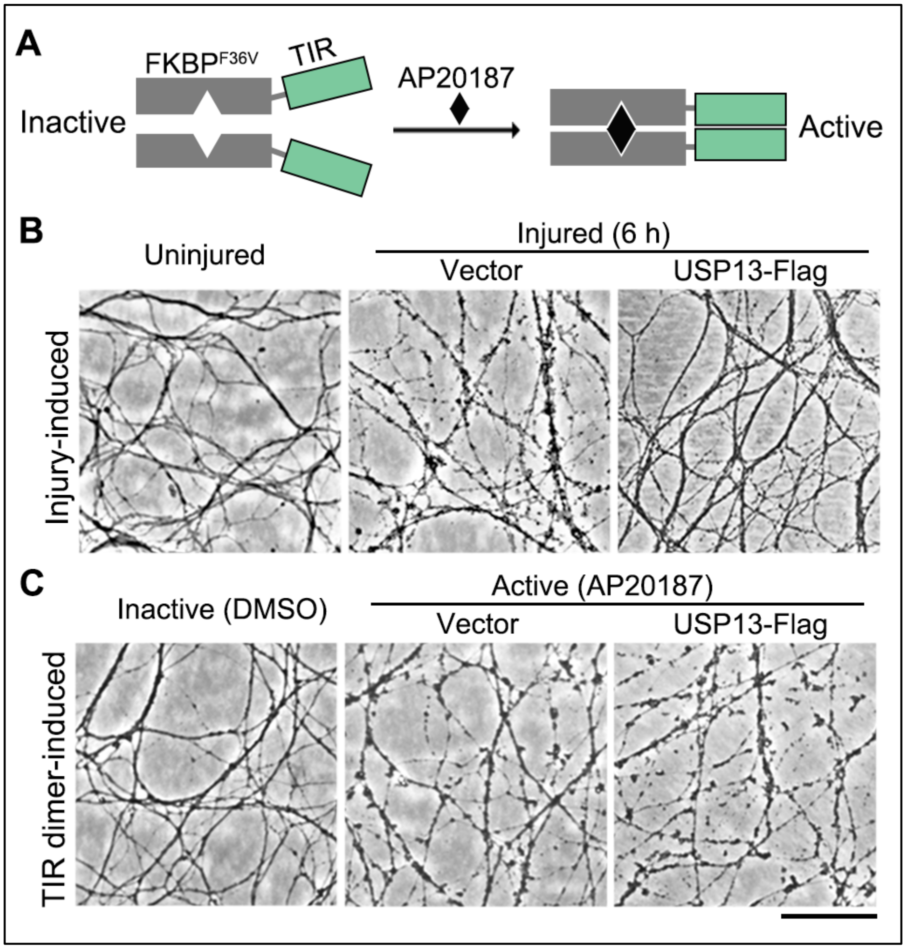

Figure S4. USP13 OE fails to suppress TIR dimerization-induced axon degeneration

(**A**) The schematic diagram of the inducible FKBP^{F36V}-TIR system. (**B-C**) Representative phase contrast images showing injury-induced (B) but not TIR dimerization-induced (100 nM AP20187, 24 h) (C) axon degeneration is delayed by USP13 OE. Scale bar: 50 μm.

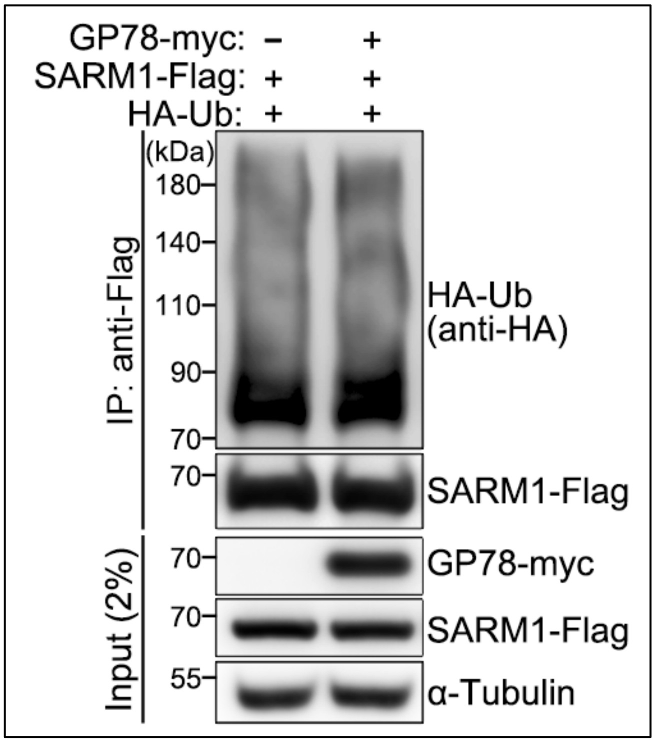

Figure S5. OE of GP78 does not affect the ubiquitination levels of SARM1

Representative western blot images showing the ubiquitination levels of SARM1 in 293T cells in the absence or presence of OE of GP78.

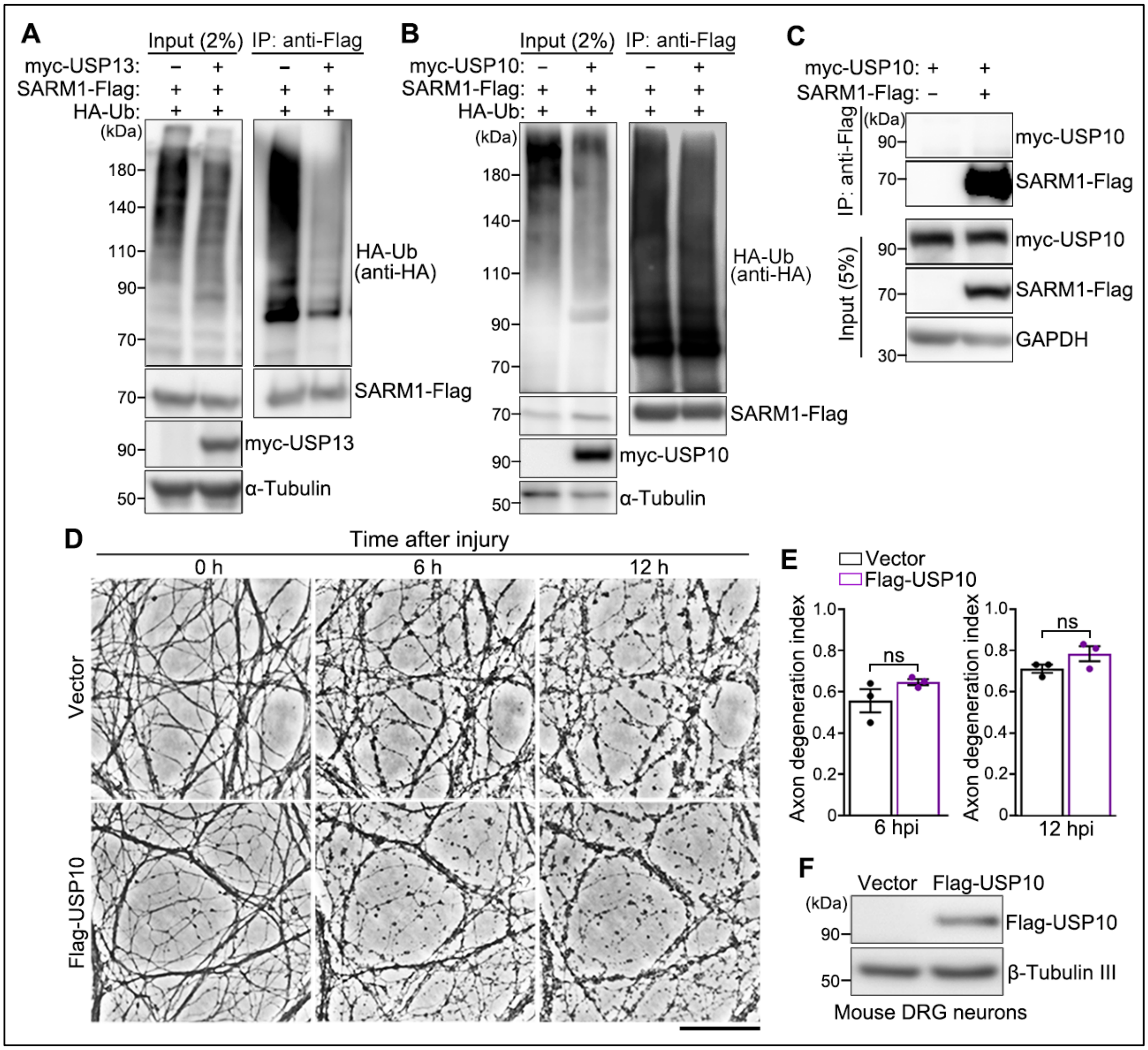

Figure S6. USP10 OE reduces the overall, but not SARM1-specific, ubiquitination levels and shows no axonal protection

(**A-B**) Western blot images showing the overall and SARM1-specific ubiquitination levels in 293T cells co-expressed with USP13 (A) or USP10 (B). (**C**) Representative images of IP and western blotting showing the interaction between SARM1 and USP10. (**D-E**) Representative phase contrast images (D) and quantified axon degeneration indexes (E) of the injured DRG neurites infected with the lentivirus expressing Flag-USP10 or the empty vector as a control. Images are

captured *live* at the indicated time points after injury. (**F**) Western blot images confirming the OE of USP10 in mouse DRG neurons. Mean ± SEM; n = 3. Student's *t*-test; ns, no significance. Scale bar: 50 μm.

SUPPLEMENTAL TABLES

Table S1. List of 73 SARM1-interacting proteins identified by the mass spec analysis

Table S2. List of 87 ARM-interacting proteins identified by the mass spec analysis

Table S3. Information of the PCR primers and siRNAs used in this study
